# Impact of differential cyclin D1 expression and localisation in prostate cancer

**DOI:** 10.1038/sj.bjc.6603615

**Published:** 2007-03-20

**Authors:** C E S Comstock, M P Revelo, C R Buncher, K E Knudsen

**Affiliations:** 1 Department of Cell and Cancer Biology, University of Cincinnati College of Medicine, Cincinnati, OH 45267, USA; 2 Department of Pathology and Laboratory Medicine, University of Cincinnati College of Medicine, Cincinnati, OH 45267, USA; 3 Department of Environmental Health, University of Cincinnati College of Medicine, Cincinnati, OH 45267, USA; 4 Center for Environmental Genetics, University of Cincinnati College of Medicine, Cincinnati, OH 45267, USA; 5 University of Cincinnati Cancer Center, University of Cincinnati College of Medicine, Cincinnati, OH 45267, USA

**Keywords:** prostate-specific antigen, Ki-67, p21^Cip1^, cytoplasmic, proliferation, androgen receptor

## Abstract

Cyclin D1 is a critical regulator of androgen-dependent transcription and cell cycle progression in prostate cancer cells. Despite the influence of D-type cyclins on prostate cancer proliferation, few studies have examined the expression of cyclin D1 in localised tumours or challenged its relevance to disease progression. Cyclin D1 status was characterised using immunohistochemistry in 38 non-neoplastic prostate samples, 138 primary human prostate carcinomas, and three lymph node metastatic specimens. Relevance of cyclin D1 to preoperative prostate-specific antigen (PSA) levels, Ki-67 index, and p21^Cip1^ status was also examined. Cyclin D1-positive phenotype was increased in primary carcinoma compared to non-neoplastic tissue, and was evident in all lymph node metastases cases. Interestingly, at least three distinct localisation patterns were observed in the cyclin D1-positive cohort, wherein cytoplasmic localisation was identified in a large fraction, and this pattern was predominant in lower grade tumours. Relevance of altered cyclin D1 status was observed, wherein cyclin D1-positive tumours were associated with low preoperative PSA levels, consistent with *in vitro* reports that cyclin D1 may alter the expression of this tumour marker. Moreover, tumours with predominantly cytoplasmic cyclin D1 showed the lowest Ki-67 index, whereas nuclear cyclin D1 was associated with higher grade, elevated Ki-67, and increased nuclear p21^Cip1^. These data demonstrate that differential cyclin D1 status may influence clinicopathological parameters, and reveal new insight as to the regulation and potential consequence of cyclin D1 expression in prostate cancer.

Prostatic adenocarcinoma is the most commonly diagnosed malignancy in the US, and is a leading cause of cancer death in men (Jemal *et al*, 2006). This tumor type is dependent on androgen for growth and survival, and treatment for metastatic disease capitalises on this dependence. Androgen elicits its biologic effects through activation of the androgen receptor (AR), a ligand-dependent transcription factor that belongs to the nuclear receptor superfamily (Trapman, 2001; Asatiani and Gelmann, 2005; Gao *et al*, 2005). Upon ligand binding, activated AR stimulates a gene expression program that induces cellular proliferation. As such, first line therapy for disseminated prostate cancer entails ablation of AR activity, either through deprivation of ligand or through the use of direct AR antagonists (Sonpavde *et al*, 2006). These therapies are highly effective, and result in tumour cell death or cell cycle arrest (Agus *et al*, 1999). Moreover, therapeutic efficacy is clinically monitored by reductions in serum PSA (prostate specific antigen), as expression of PSA is dependent on AR activity in prostatic cells and correlates with tumour burden (Catalona and Loeb, 2005). Despite the initial success of therapy, recurrent tumours ultimately form wherein AR activity has been restored, and this event is typically preceded by a detectable rise in serum PSA (Feldman and Feldman, 2001). Restoration of AR activity is known to occur through several discrete pathways, and in model systems of prostate cancer reactivation of AR is causative for therapeutic relapse and tumour recurrence (Chen *et al*, 2004). Moreover, the majority of studies have shown that recurrent, androgen-independent prostate cancer cells still require AR activity for proliferation and survival (Taplin and Balk, 2004; Haag *et al*, 2005).

Given the importance of androgen action and AR function in prostate cancer growth and progression, much emphasis has been devoted to delineating the mechanisms by which AR promotes tumour cell proliferation and the factors that govern these events. Toward this end, we and others have shown that as part of its proproliferative program, androgen induces accumulation of D-type cyclins (Knudsen *et al*, 1998; Xu *et al*, 2006). This class of cyclins interact with and activate cyclin-dependent kinases (CDK) 4/6 to promote G_1_ progression during the cell cycle (Mittnacht, 1998; Sherr and Roberts, 2004). Recent studies have shown that AR stimulates cell cycle progression at least in part through induction of mTOR (mammalian Target of Rapamycin) activity, which facilitates cyclin D protein accumulation (Xu *et al*, 2006). Additional factors are also suspected to control this event, such as p21^Cip1^, which is an essential accessory factor for the assembly, stabilisation, and translocation of active cyclin D1/CDK4 complexes to the nucleus (Cheng *et al*, 1999; Parry *et al*, 1999). Furthermore, p21^Cip1^ protein levels are induced by androgen stimulation (Knudsen *et al*, 1998) and p21^Cip1^ has been shown to be a direct AR target gene (Lu *et al*, 1999). Displacement of p21^Cip1^ from active cyclin D1/CDK4 complexes allows p21^Cip1^ to bind and inhibit CDK2 activity, thereby inhibiting progression through G_1_ and into S phase (Sherr and Roberts, 2004). Thus, the ability of cyclin D1 to interact with p21^Cip1^ is thought to be a critical function in promoting cellular proliferation and is often associated with mitogenic stimulation (Alt *et al*, 2002).

In prostate cancer cells, it has been shown that cyclin D1 induction is insufficient to drive androgen-independent proliferation (Knudsen *et al*, 1998; Fribourg *et al*, 2000), and forced elevation of cyclin D1 in the presence of androgen suppresses rather than promotes cellular proliferation (Petre-Draviam *et al*, 2003). These effects are at least partially attributed to the ability of cyclin D1 to bind and inhibit AR activity (Knudsen *et al*, 1999; Reutens *et al*, 2001), as achieved through at least two discrete mechanisms. First, cyclin D1 binds an N-terminal region of AR that is required for docking to its C terminus upon ligand binding and suppresses the efficacy of this N–C interaction (Burd *et al*, 2005). Second, cyclin D1 can associate with histone deacetylases to repress transcription, and this function of cyclin D1 is essential for its AR corepressor activity (Lin *et al*, 2002; Petre-Draviam *et al*, 2005). Cyclin D1 can also bind to and modulate other transcription factors by similar mechanisms, with the largest class of proteins belonging to the nuclear receptor superfamily (Coqueret, 2002; Ewen and Lamb, 2004; Fu *et al*, 2004). These collective observations have culminated in a model whereby androgen-mediated induction of cyclin D1 serves to activate CDK4 and promote cell cycle progression, but that accumulated cyclin D1 engages in a negative feedback loop to limit or modulate the response to androgen stimulation. In the prostate, it is proposed that cyclin D1 acts as a rheostat to control the strength and duration of androgen stimulation and AR activity.

Despite the importance of cyclin D1 in eliciting and modulating the androgen response in prostate cancer, few studies have examined the expression profile of this protein in localised tumours or evaluated its relevance for disease progression. We examined 138 human radical prostatectomy samples for cyclin D1 expression, as compared to normal prostatic epithelia. We show that whereas cyclin D1 expression is low or absent in non-neoplastic tissue, its levels are increased in the majority of localised tumours. Surprisingly, four distinct expression profiles were observed in these tumour sets, wherein a large fraction of cyclin D1-positive tumours showed cytoplasmic restriction. Expression profiles showed some grade specificity, wherein nuclear cyclin D1 staining emerged almost exclusively in the higher grade tumours. Additionally, PSA expression was lower in the cyclin D1-positive tumours, indicating that cyclin D1 status may affect expression of serum markers that are dependent on AR activity. The relevance of cyclin D1 status to the proliferative index was also considered, wherein tumours with predominantly cytoplasmic cyclin D1 exhibited the lowest proliferative index, even as compared to cyclin D1-negative tumours. Lastly, nuclear p21^Cip1^ status was investigated, and p21^Cip1^ levels frequently associated with a more proliferative and predominantly nuclear cyclin D1 phenotype. Together, the data herein is the first to demonstrate that cyclin D1 can be differentially expressed in prostate cancer, and that the status and/or localisation of cyclin D1 expression are associated with meaningful changes in tumour marker expression and proliferative indices.

## MATERIALS AND METHODS

### Tissue procurement

Formalin-fixed paraffin-embedded serial sections (5 *μ*m) were obtained from 36 patients that were diagnosed with prostate cancer and underwent radical prostatectomy at the University of Cincinnati Hospital between 2000 and 2004 in accordance with institutional review board standards. Adjacent, non-neoplastic tissue was associated with 23 samples and preoperative PSA values were available for 20 patients. Additionally, three separate lymph node samples with metastatic prostate cancer were obtained. Human prostate tissue microarray slides containing individual tumour cores (1.5 mm diameter, 5 *μ*m) were purchased. One array (TA1) contained 80 cores (PR801; US Biomax, Ijamsville, MD, USA), whereas the other array (TA2) contained 49 cores with nine-matched normal tissue and 35 associated PSA values (IMH-303; Imgenex, San Diego, CA, USA). All patient tumours and tissue microarrays were evaluated and graded by a pathologist according to established guidelines (Epstein *et al*, 2006).

### Immunohistochemistry

Immunohistochemical staining for cyclin D1 and Ki-67 was performed using a BenchMark automated stainer (Ventana, Tucson, AZ, USA) using the avidin–biotin peroxidase technique following standard protocols. Staining for cyclin D1 was performed, on all samples, according to manufacturer specifications (P2D11F11; Ventana, Tucson, AZ). Staining for Ki-67 (1 : 25, MIB-1; DAKO, Carpinteria, CA, USA) was performed using a subset of 22 samples from the 36 patient samples and one tissue microarray (TA1). Positive and negative controls for both antibodies gave the expected results. Immunohistochemistry for p21^Cip1^ (1 : 1000, SC-397; Santa Cruz, CA, USA) and AR (1 : 5000, SC-816; Santa Cruz, CA, USA) was performed using a Vectastain Elite ABC rabbit staining kit according to manufacturer specifications (Vector Laboratories, Inc., Burlingame, CA, USA). Briefly, a subset of 16 patient samples and all three lymph node metastatic samples were de-paraffinised in xylene and rehydrated to 70% ethanol. Antigen retrieval was achieved in a 600 W microwave using an antigen unmasking solution (Vector Laboratories, Inc., Burlingame, CA, USA). Following staining, the antigen was visualised using diaminobenzidine substrate for peroxidase using a 2 min development (DAKO, Carpinteria, CA, USA) and counterstained with haematoxylin.

### Scoring and statistical analysis

Nuclear and cytoplasmic cyclin D1 staining intensity was assessed before the clinical parameters (MPR) and semi-quantitatively scored as 0 (absent), 1- (weak/focal <10% of sample), 1+ (weak intensity <25% of sample), 2+ (moderate intensity 25–50% of sample), or 3+ (strong intensity >50% of sample). The percentage of nuclear Ki-67 and p21^Cip1^ were counted without knowledge of the clinical data, using a 10 × 10 grid eyepiece at × 20 magnification. Prostate and metastatic tumour samples were assessed by counting nine separate fields, whereas only five separate fields could be counted for each tumour core on the microarray. Approximately 600 cells were counted in each field. The data were plotted by Graph Pad Prism (v4.0) using the mean±standard error (s.e.m.) or by scatter plot with the mean value. Statistical analysis was also performed and correlations were determined by linear regression. Significant differences (*P*<0.05) between multiple groups were determined by one-way analysis of variance using Kruskal–Wallis with a Dunn's multiple comparison *post hoc* test of all pairwise comparisons, or by two-tailed *t*-test for two group comparisons.

## RESULTS

### Disparate localisation of cyclin D1 is associated with prostate tumour grade

**Figure 1:**
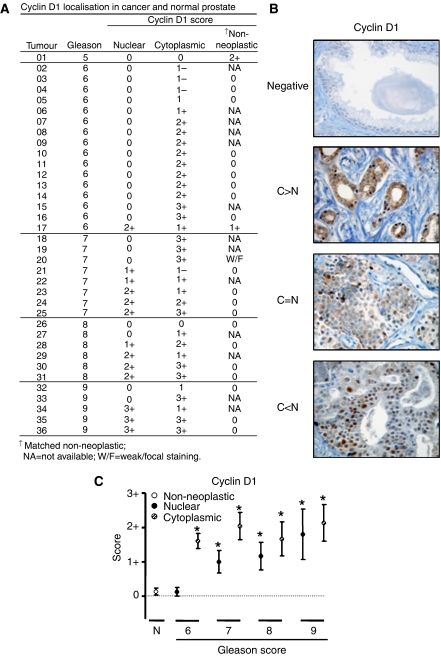
Disparate localisation of cyclin D1 in localised prostate carcinoma is tumour grade specific. (**A**). Summary of cyclin D1 immunohistochemistry in human primary prostate tumours (*n*=36). Tumours are grouped according to Gleason and cyclin D1 scores. (**B**). Representative cyclin D1 immunohistochemical images (×40 magnification) from primary prostate tumours displaying four different localisation patterns: negative, higher cytoplasmic than nuclear (C>N), cytoplasmic equal to nuclear (C=N), and higher nuclear than cytoplasmic (C<N). (**C**). Non-neoplastic (○), nuclear (•), and cytoplasmic (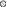) cyclin D1 scores for each Gleason grade were averaged from Figure 1A, and presented as mean±s.e.m. Only values with more than one determination are shown. Statistical differences from non-neoplastic are indicated (^*^) and described in the Results section.

To dissect the expression patterns of cyclin D1 in prostate cancer, an initial cohort of 36 human primary prostate adenocarcinomas was examined by immunohistochemistry. Of these, matched non-neoplastic adjacent tissues were available for 23 specimens. The Gleason and cyclin D1 scores were assessed for each patient specimen (Figure 1A). In agreement with the literature (Han *et al*, 1998; Aaltomaa *et al*, 1999; Murphy *et al*, 2005), cyclin D1 was absent in the majority of non-neoplastic prostate tissue (20/23) and of the three positive cases one exhibited weak/focal staining. These data are consistent with the low proliferative index of adult prostatic epithelia. Interestingly, of the cyclin D1-positive tumours (34/36), disparate cyclin D1 staining was observed in the tumour regions. Staining was confined to the epithelial-derived tumour cells, and stromal regions were negative in all cases. At least four distinct cyclin D1 expression patterns were identified (Figure 1B). First, the majority of tumours scored high for cytoplasmic cyclin D1 with little or no nuclear cyclin D1 reactivity (25/36). Second, a minority of tumours showed predominately nuclear cyclin D1 (5/36). Third, a similar number of cases showed equivalent cyclin D1 staining in both the nuclear and cytoplasmic compartments (4/36). The fourth pattern was represented by those tumours devoid of cyclin D1 (2/36). These data are consistent with the *in vitro* observations that cyclin D1 can be stringently regulated as a function of subcellular localisation (Alt *et al*, 2000; Radu *et al*, 2003) and reveal that distinct localisation patterns are observed in prostate cancer.

Previous studies have shown that cyclin D1 activity is strongly influenced by its subcellular localisation (Diehl and Sherr, 1997; Barr and Johnson, 2001; Lin and Gelman, 2002; Balasenthil *et al*, 2004; Alao *et al*, 2006; Sumrejkanchanakij *et al*, 2006). Given the distinct patterns of localisation observed in the initial cohort of prostatectomy specimens, the prevalence of cyclin D1 staining patterns was examined as a function of tumour grade. Cytoplasmic cyclin D1 staining was significantly increased in tumours compared to non-neoplastic tissue (p<0.05) and appeared to be predominant in Gleason 6 and 7 tumours (Figure 1C). By contrast, nuclear cyclin D1 emerged in higher grade tumours (Gleason 8 and 9) and was also accompanied by high cytoplasmic cyclin D1 staining. Importantly, a positive linear association between Gleason score and nuclear cyclin D1 was observed (*P*<0.01). These data indicate that disparate cyclin D1 staining patterns correlated with specific tumour grades.

To challenge further these observations, the initial cohort was expanded to include two different human prostate carcinoma tissue microarrays (Table 1, TA1 and TA2). For clarity, the cases are presented separately by cohort and annotated by Gleason score and cyclin D1 localisation pattern. As shown, the four identified staining patterns observed in the initial cohort (Figure 1A) were also observed in each tissue microarray. As expected, all 15 non-neoplastic cases were cyclin D1-negative. Of the combined 102 tumour specimens, the largest fraction scored negative for cyclin D1 (40 and 62.2%; TA1 and TA2, respectively) suggesting that cyclin D1 expression is not requisite for tumour maintenance. Cytoplasmic restriction of cyclin D1 staining was also observed (9.2 and 16.2%, TA1 and TA2, respectively), and this class was associated with the lower Gleason scores. As in the initial cohort, tumours in the microarray with a high Gleason score maintained, cytoplasmic cyclin D1, but tended to be accompanied by higher nuclear cyclin D1. Combined, these data provide evidence that cyclin D1 expression is increased and shows distinct localisation patterns in localised disease, wherein these patterns are altered as a function of Gleason score.

### Cyclin D1 expression in metastatic prostate carcinoma

**Figure 2:**
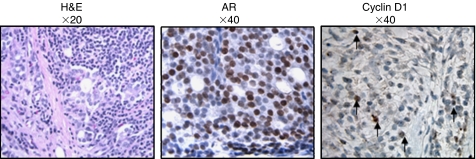
Cyclin D1 expression in metastatic prostate carcinoma. Representative immunohistochemical images from prostate-derived lymph node metastatic tumours. *Left panel*: Haematoxylin and eosin staining showing disorganised architecture of the metastatic tumours. *Middle panel*: Strong nuclear positive AR staining in the majority of tumour cells. *Right Panel).* Low nuclear cyclin D1 staining (score of 1+). Arrows indicate tumours cells with evident positive staining. Magnification: Left (H&E), × 20; middle and right (Haematoxylin counterstained), × 40.

The unique patterns of cyclin D1 in localised tumours prompted us to investigate cyclin D1 expression in metastatic tumours. For these studies, cyclin D1 localisation was evaluated in lymph node metastases obtained from three individual patients. Haematoxylin and eosin (H&E) staining of all three cases revealed a similar morphology with densely packed and disorganised tumour cells and no glandular structure (Figure 2, left panel and data not shown). All three metastatic tumours stained nuclear positive for the AR, thus validating the tumour cells as prostatic in origin (Figure 2, middle panel and data not shown) Although the available sample size is small, all tumours exhibited exclusively modest (1+) nuclear cyclin D1 staining (Figure 2, right panel and data not shown), whereas the surrounding stromal tissue was null for cyclin D1. These results suggest that cyclin D1 localization may be altered in lymph node metastases, wherein cyclin D1 expression is low and confined to the nucleus.

### Cyclin D1 status is inversely associated with PSA levels

**Figure 3:**
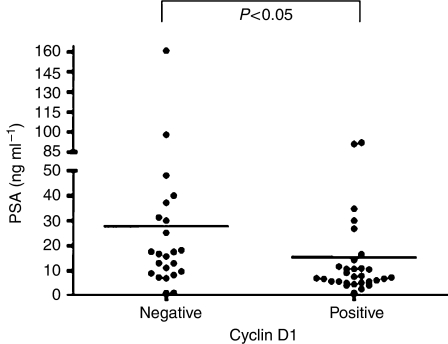
Cyclin D1 status inversely associates with PSA levels. Combined preoperation PSA values (ng ml^−1^) separated as negative (*n*=24) and positive (*n*=29) for cyclin D1 and presented as a scatter plot. Average preoperative PSA values for the cyclin D1-positive and -negative tumours were statistically different (*P*<0.05). Average values for each group are described in the Results section.

Cyclin D1 has the potential to regulate both cellular proliferation and AR-dependent transcription in prostate cancer cells (Burd *et al*, 2006a). Therefore, a link between the expression of cyclin D1 and PSA, an AR target gene, was examined. For these studies, the impact of cyclin D1 status on AR function was assessed using preoperative PSA levels. Of all the tumours examined, preoperative PSA levels were available for 53 patients. From the available specimens, 23 (43.3%) were cyclin D1-negative and 30 (56.6%) were cyclin D1-positive (Figure 3). As shown, the mean PSA value for the cyclin D1-positive group (15.5±4.03 ng ml^−1^) was significantly lower than the cyclin D1-negative group (27.7±7.43 ng ml^−1^) (*P*<0.05). Unfortunately, there was insufficient statistical power to separate the PSA values according to cyclin D1 localisation or Gleason score (data not shown). However, these data are consistent with the established ability of cyclin D1 to suppress AR function and suggest that cyclin D1 status may influence factors relevant to prostate cancer diagnosis.

### Cyclin D1 localisation impacts proliferative status in prostate carcinoma

**Figure 4:**
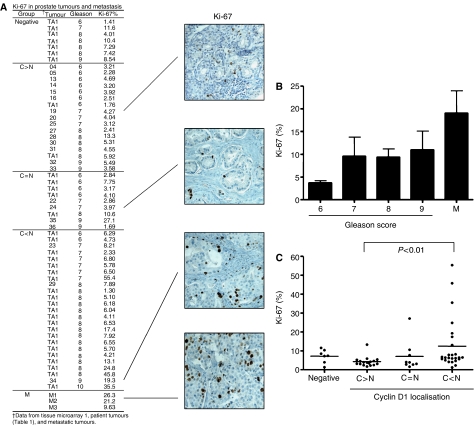
Cyclin D1 status impacts proliferation in prostate carcinoma. (**A**) Summary of nuclear Ki-67 (%) immunohistochemistry for a subset of patient tumours (*n*=22), a tissue microarray (TA1, *n*=36), and the metastatic tumours (*n*=3). Tumours are grouped according to cyclin D1 localisation as described in Figure 1B and ranked by Gleason grade. (**B**) Histograph of Ki-67 (%) as a function of tumour progression (*n*=22); the data (Mean±s.e.m.) were derived from Figure 1A. A positive correlation between tumour progression and Ki-67 (%) was seen (*P*<0.05). (**C**) Ki-67 (%) were separated according to cyclin D1 localisation and presented as a scatter plot. *Note*: A number of D1-positive tumours in all the three localisation groups demonstrated low proliferation. The average Ki-67 (%) for C>N was statistically different than C<N (*P*<0.01). Average values for each group are described in the Results section.

The ability of cyclin D1 to control transcription factor activity is appreciated in a multitude of tissues. However, the canonical role of cyclin D1 is in its ability to promote cellular proliferation. The relevance of cyclin D1 localisation, in particular cytoplasmic localisation, to proliferative status has not been evaluated. To test this, representative tumours from each cyclin D1 staining category were processed to quantify the proliferative indices by Ki-67 immunoreactivity. Specific staining for Ki-67 was indicated by the absence of immunoreactivity in the adjacent matched-normal tissue or in the stromal regions. The raw Ki-67 data with representative images are provided in Figure 4A, and grouped according to cyclin D1 localisation and Gleason score. A scatter analysis between Gleason score and Ki-67 status was assessed in the patient tumours and tissue microarray, and regression analysis was similar for each group (data not shown). These data are consistent with previous reports (Baretton *et al*, 1999) and provided confidence in the combined dataset. As expected, a significant positive correlation between localised tumour score and proliferation was determined (*P*<0.05), and the highest average proliferative index came from the metastatic tumours (Figure 4B). When the impact of cyclin D1 localisation on proliferation was examined (Figure 4C), a statistically significant (*P*<0.01) difference was identified between predominately cytoplasmic cyclin D1 (4.33±0.63) and predominately nuclear cyclin D1 (12.5±2.77) tumours. The proliferative status of the cytoplasmic cyclin D1 tumours were typically lower than the cyclin D1-negative (7.25±1.34) tumours, although this did not reach statistical significance. This potentially interesting observation may suggest that cytoplasmic localisation may play a role in suppressing cellular proliferation. Furthermore, these data support the role of nuclear cyclin D1 as an initiator of cell cycle progression, where it is able to bind and activate CDK. However, it should be noted that a number of cyclin D1-positive tumours in all three localisation groups had a low proliferative index (Figure 4C), demonstrating that cyclin D1 expression itself is not sufficient to induce cellular proliferation.

### Contribution of p21^Cip1^ to proliferation and progression of prostate carcinoma

**Figure 5:**
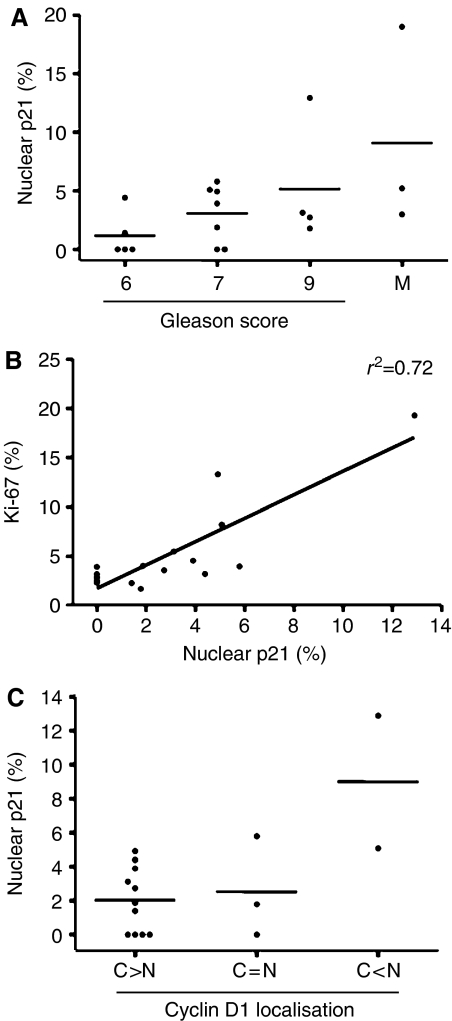
Contribution of p21^Cip1^ expression in prostate carcinoma. Scatter plots characterising nuclear p21^Cip1^ (%) in a subset of patient tumours (PT1, *n*=16), which had been immunostained for cyclin D1 and Ki-67. (**A**) A trend between increased nuclear p21^Cip1^ and Gleason score was observed. (**B**) A positive correlation by linear regression was identified between increased nuclear p21^Cip1^ and Ki-67 (%) (*r*^2^=0.72). (**C**) Tumours with more nuclear D1 appear to have more nuclear p21^Cip1^. Average values for panels A and C are described in the Results section.

Although the mechanisms governing cyclin D1 localisation are not completely understood, p21^Cip1^ is a key modulator of both cyclin D1 activity and its subcellular localisation. Interaction of p21^Cip1^ with cyclin D1/CDK is known to promote stabilisation and nuclear import of the complex, resulting in enhanced CDK activity (Parry *et al*, 1999; Gladden and Diehl, 2005). Similarly, p21^Cip1^ has been shown to be increased in prostate cancer and holds potential merit as a prognostic indicator (Matsushima *et al*, 1998; Sarkar *et al*, 1999; Omar *et al*, 2001; Rigaud *et al*, 2004). To determine the relevance of p21^Cip1^ to cyclin D1 localisation and proliferation, p21^Cip1^ immunoreactivity was assessed in a subset of patient tumours (*n*=16) and the three metastatic tumours wherein cyclin D1 and Ki-67 status had been determined. All three metastatic tumours and 11 of the 16 primary tumours (68.8%) were p21^Cip1^-positive. The range of p21^Cip1^ staining was between 1.41–12.9% of the tumour specimens. A positive trend was observed between tumour grade and nuclear p21^Cip1^, but these data did not reach statistical significance (Figure 5A). However, a significant positive correlation was identified between nuclear p21^Cip1^ and Ki-67 with an *r*^2^ of 0.72 (Figure 5B). Separation by cyclin D1 localisation indicated that samples with higher nuclear cyclin D1 are likely to have higher nuclear p21^Cip1^ (Figure 5C), as the mean percent nuclear p21^Cip1^ for the cyclin D1 groups were: C>N (2.04±0.57), C=N (2.53±1.71), and C<N (8.99±3.91). However, no obvious trends were identified between p21^Cip1^ and PSA or Gleason score (data not shown). Together, these data suggest that p21^Cip1^ levels likely contribute more towards nuclear than cytoplasmic localisation of cyclin D1, and that increased nuclear p21^Cip1^ levels may be associated with a more proliferative or advanced disease.

## DISCUSSION

It has been previously shown through *in vitro* studies that cyclin D1 can influence androgen-dependent prostate cancer cell proliferation through its dual ability to modulate both CDK4 and AR activity (Petre-Draviam *et al*, 2005; Burd *et al*, 2006a). To elucidate further the role of cyclin D1 in prostate cancer, primary human prostate cancer specimens were utilised to assess the influence of cyclin D1 expression and localisation on PSA levels and proliferation index. The present findings demonstrate that cyclin D1 expression is enhanced in the majority of localised tumours as compared to non-neoplastic epithelia, thus indicating that cyclin D1 is aberrantly regulated in prostate cancer. Remarkably, cyclin D1-positive tumours displayed distinct localisation patterns, wherein tumours frequently exhibited predominately cytoplasmic cyclin D1. Investigations to challenge the impact of cyclin D1 expression revealed that cyclin D1-positive tumours associated with significantly lower preoperative PSA values, indicating that cyclin D1 status may influence tumour marker expression. Furthermore, there was a trend for tumours with predominately cytoplasmic cyclin D1 to harbour low proliferative potential as compared to tumours with predominately nuclear cyclin D1. Finally, expression of nuclear p21^Cip1^, an important mediator of cyclin D1 action, correlated with proliferation and was associated with predominately nuclear cyclin D1, thus providing a potential mechanism for the differential localisation patterns of cyclin D1. Combined, these data suggest that cyclin D1 expression and localisation may influence proliferation and diagnostic factors in prostate cancer.

Few studies have addressed cyclin D1 expression or localisation in primary prostatic adenocarcinomas, and the criteria used to establish positive cyclin D1 staining have been divergent. The majority of studies have focused largely on nuclear cyclin D1, but common conclusions have failed to emerge. For example, using a staining cutoff of >10%, a trend for increased nuclear cyclin D1 with high Gleason scores (⩾7) was observed but not considered significant (Kallakury *et al*, 1997). A separate study, using a low (<20%) -*vs*- high (>20%) nuclear cyclin D1 staining criteria, reported only 12% of primary tumours with high cyclin D1 (Drobnjak *et al*, 2000). In the present analyses, quantification of all cyclin D1 localisation patterns was considered. Using this inclusive approach, 63% of tumours and only 8% of normal epithelia scored positive for cyclin D1 (Table 1), indicating that the protein is accumulated in prostate cancer. These data are congruent with a large study of 187 tumours, wherein 71% were scored cyclin D1-positive, with some cytoplasmic cyclin D1 noted but not quantified (Aaltomaa *et al*, 1999). Here, nuclear cyclin D1 correlated with Gleason score and proliferation, as is also consistent with our results (Figures 1C and 4C, respectively). A series of smaller studies have also reported cyclin D1 induction in tumours compared to normal specimens (Han *et al*, 1998; Kolar *et al*, 2000; Murphy *et al*, 2005) and at least one of these considered all cyclin D1 localisations (including cytoplasmic and perinuclear) as positive, although correlates according to localisation were not considered (Han *et al*, 1998). Thus, the data herein confirm that increased cyclin D1 is seen with high frequency in prostate cancer, and is the first to provide a detailed assessment of cyclin D1 localisation patterns and correlates in organ-confined disease.

The impact of cyclin D1 localisation patterns in metastatic disease is less certain. This study examined only three prostate-derived metastatic tumours, all of which showed low nuclear cyclin D1 staining and lacked cytoplasmic staining (Figure 2). Consistent with these results, previously reported data have shown that 84% of lymph node metastases lack significant cyclin D1 staining (Kallakury *et al*, 1997). In contrast, a second study assessed nuclear cyclin D1 in bone metastatic tumours and determined that a large fraction (68%) of these specimens contained cyclin D1 (Drobnjak *et al*, 2000). These data suggest that cyclin D1 may hold a more pronounced role for proliferation in bone metastases compared to lymph node deposits. Consistent with a role for cyclin D1 in the proliferation of bone metastatic tumours from prostate, others have reported correlations between metastatic cyclin D1 expression derived from other tumour types (Chhieng *et al*, 1998). Given these observations, the concept that cyclin D1 prevalence and localisation may vary depending on metastatic site is intriguing and should be more rigorously considered.

Regardless of the site, the mechanism(s) by which cyclin D1 accumulates in prostate cancer cells remains to be discerned. Cyclin D1 levels are typically under stringent regulation, but aberrant cyclin D1 expression has been implicated as having oncogenic properties in many neoplasias including breast (Arnold and Papanikolaou, 2005), pituitary (Faglia and Spada, 2001), and squamous cell carcinoma (Nadal and Cardesa, 2003). Overexpression of cyclin D1 may occur through multiple mechanisms, including chromosomal translocation events (e.g. Mantle cell lymphomas (Bigoni *et al*, 1996)), gene amplification (common in breast (Buckley *et al*, 1993) and oesophageal cancers (Jiang *et al*, 1993)), and defects in protein processing or transport (Gladden and Diehl, 2005). The propensity of selected tumour types to induce cyclin D1 accumulation in cancer may therefore be a heterogenous and complex event that culminates in a similar phenotype. Although genetic abnormalities in prostate cancer have been reported for other important cell cycle regulators such as phosphatase and tensin homologue (Majumder and Sellers, 2005), the retinoblastoma tumour suppressor protein (RB), and p53 (MacGrogan and Bookstein, 1997), perturbations in cyclin D1 appear to be less common. For example, cyclin D1 gene amplification did not increase in benign prostatic hyperplasia (BPH) or primary and refractory tumours of the prostate (Linja *et al*, 2004). Similarly, others have shown low cyclin D1 gene amplification (4%) in primary prostate tumours (Gumbiner *et al*, 1999) and, depending on the method of detection, 5–15% gene amplification in advanced prostate tumours (Bubendorf *et al*, 1999; El Gedaily *et al*, 2001). Thus, it is unlikely that the induction of cyclin D1 observed herein is a result of gene amplification. Recent studies in prostate cancer model systems have shown that cyclin D1 accumulates after androgen stimulation as a result of mTOR activation and resultant enhancement of cyclin D1 mRNA translation (Xu *et al*, 2006). However, the mechanisms underlying androgen-mediated mTOR induction remain elusive. Future studies directed at delineating mTOR activation may assist in revealing the mechanism(s) by which cyclin D1 accumulates in prostate cancer.

Once cyclin D1 protein accumulates, it is often regulated by subcellular localisation and proteasome-dependent degradation (Diehl *et al*, 1998; Germain *et al*, 2000). Our data indicate that this process, in part, is likely to be responsible for the disparate localisation of cyclin D1 in prostate cancer. The present finding that prostate cancer cells frequently exhibit cytoplasmic cyclin D1 is novel, but not without precedent. For example, elevated cyclin D1 with cytoplasmic cyclin D1 staining has been reported in many different tumour types, such as colorectal (Handa *et al*, 1999; Khor *et al*, 2006), pancreatic (Culhaci *et al*, 2005), lung (Dworakowska *et al*, 2005), thyroid (Temmim *et al*, 2006), and bladder (Sudo *et al*, 2003). Despite these observations only a handful of studies have addressed the functional consequence of cytoplasmic cyclin D1. It has been suggested that cytoplasmic cyclin D1 is more common in colorectal tumours deficient for *β*-catenin (Kuramochi *et al*, 2006); however, *β*–catenin has been shown to regulate cyclin D1 expression in colon cancer cells (Tetsu and McCormick, 1999). In pituitary (Hibberts *et al*, 1999; Simpson *et al*, 2001) and ovarian (Dhar *et al*, 1999) tumours, cytoplasmic cyclin D1 has been reported (35 and 59% of cyclin D1-positive cases, respectively) but no correlations were identified. In hepatocellular carcinomas, cytoplasmic cyclin D1 appeared to significantly correlate with prognostic factors like intrahepatic metastasis (Sato *et al*, 1999). A large study which assessed cyclin D1 localisation was performed with 150 transitional cell carcinomas of the bladder, wherein cytoplasmic cyclin D1 increased with disease progression and correlated with decreased survival (Tut *et al*, 2001). This contrasts with our findings, wherein predominately cytoplasmic cyclin D1 correlated with lower Gleason scores (Figure 1C and Table 1) and exhibited the lowest proliferation index (Figure 4C). Furthermore, the proliferative index tended to be lower in the cytoplasmic cyclin D1 tumours than in the cyclin D1-deficient tumours. This observation suggests that restriction of cyclin D1 to the cytoplasm may allow suppression of cellular proliferation but requires a more rigorous investigation. Interestingly, a number of colorectal cancer studies have observed cytoplasmic cyclin D1 in tumour specimens (Palmqvist *et al*, 1998; Handa *et al*, 1999; McKay *et al*, 2000; Holland *et al*, 2001; Khor *et al*, 2006; Kuramochi *et al*, 2006) and at least one of these studies has reported that increased cytoplasmic cyclin D1 correlated with low proliferation (Palmqvist *et al*, 1998). This result is similar to our analyses (Figure 4C), wherein tumours with predominately cytoplasmic cyclin D1 showed a significantly lower proliferative index. In the case of prostate cancer, one mechanism could be through the ability to directly repress AR activity or sequester it from its nuclear targets. Alternatively, prostate cancer cells may have invoked mechanisms to bypass the cyclin D1 requirement (e.g. RB loss), thereby weakening the necessity to induce cyclin D1 accumulation (Lukas *et al*, 1995). However, it should be emphasised that our data also suggests that cyclin D1-positive status does not predict a high proliferative index (Figure 4C). Combined, these data support the contention that cyclin D1 is differentially localised in cancer, and that cytoplasmic cyclin D1 can correlate with proliferative outcomes in a tissue specific manner.

The emergence of nuclear cyclin D1 in the high Gleason scores is intriguing, and this localisation pattern correlated with a higher proliferative index. These observations are consistent with the ability of nuclear cyclin D1 to enhance CDK4 activity and promote G_1_-S progression. The delineation of the mechanism(s) by which nuclear emergence is facilitated will likely reveal new insights into advanced prostatic disease. One exciting possibility to be considered concerning nuclear cyclin D1 is the contribution of cyclin D1b, a splice variant of cyclin D1 that is restricted from nuclear export (Lu *et al*, 2003). Attempts to develop a suitable cyclin D1b-specific antibody for immunohistochemistry have had limited success (data not shown). However, we have recently shown that cyclin D1b is upregulated in human prostate tumours, is selectively compromised for AR regulation, and may yield a specific growth advantage (Burd *et al*, 2006b). Therefore, future investigations will be directed at assessing cyclin D1b in tumours as a function of disease progression.

In addition to the effects on proliferation, previous data in prostate cancer cell lines suggest that cyclin D1 has specialised roles concerning AR function in the control of prostate cancer cellular function. Supporting this view, the present analyses revealed that cyclin D1 status can significantly correlate with serum PSA levels (Figure 3). Specifically, cyclin D1-positive tumours were more likely to be derived from patients with lower preoperative PSA values, perhaps reflective of the established ability of cyclin D1 to modulate AR function. This contention is consistent with previous reports, which observed a correlation between low cyclin D1 and elevated preoperation PSA values (>10 ng ml^−1^) (Drobnjak *et al*, 2000). On the basis of the PSA and proliferation data obtained in our study, it is conceivable that a subset of cyclin D1-positive tumours may exist that have increased proliferation and have low PSA values. The mechanism(s) and consequences of this observation will be of significant clinical interest, as these data indicate that cyclin D1 status may suppress tumour marker expression. Moreover, these data highlight the need to consider the effects, mediated by regulatory elements of proliferation (i.e. cyclin D1), on tumour marker expression.

As these collective findings indicated that cyclin D1 status and localisation patterns may impinge upon critical parameters, potential mechanism(s) underlying the differential cyclin D1 patterns were investigated. Here we show that p21^Cip1^, which has been shown to positively regulate cyclin D1 accumulation, nuclear translocation, and CDK4 activation, is likely to contribute to the differential patterns observed. Specifically, we showed a trend for increased p21^Cip1^ levels with tumour progression and that p21^Cip1^ is potentially elevated in tumours with high nuclear cyclin D1 (Figure 5A and C). These observations are consistent with observations in other tumour types (McKay *et al*, 2000; Holland *et al*, 2001; Tut *et al*, 2001) and support its role in promoting cyclin D1/CDK4 activity. Second, we showed that p21^Cip1^ levels correlate with proliferation (Figure 5B), thus confirming other reports (Ljung *et al*, 1997; Baretton *et al*, 1999; Osman *et al*, 1999; Fizazi *et al*, 2002). Collectively, these data indicate that p21^Cip1^ action in this tumour type likely favours proliferation. Similar conclusions about the role of p21^Cip1^ have been seen in other tumour types such as breast cancer (Elledge and Allred, 1998). These studies indicate that p21^Cip1^ is a likely effector of cyclin D1 accumulation, localisation, and activity in multiple tumour types, including prostate.

In summary, the data herein demonstrate that whereas cyclin D1 is generally elevated in localised prostate cancer, differential localization of cyclin D1 may influence clinicopathological parameters. The present data support a model wherein cyclin D1 accumulation and localisation are regulated as a function of tumour grade, and that differential cyclin D1 status significantly correlates with proliferation and PSA values. Further analyses revealed that p21^Cip1^ may play a role in regulating cyclin D1 dynamics and likely contributes to the observed alterations in tumour characteristics. Together, these studies associated differential expression and localisation of cyclin D1 with important clinicopathological parameters and suggest new insight concerning the potential consequence of cyclin D1 induction in prostate cancer.

## Figures and Tables

**Table 1:** Immunohistochemical summary of cyclin D1 in tissue microarrays

			**Cyclin D1 localisation**		
	***n***	**Cyclin D1 negative**	**C>N^a^**	**C=N**	**C<N**
		***TA1, TA2* b**			
**Normal^c^**	**15**	**6, 9**	**—**	**—**	**—**
**Tumour** *Gleason*					
6	34	8, 13 (61.7)^d^	1, 3 (11.8)	4, 1 (14.7)	2, 2 (11.8)
7	25	4, 4 (32.0)	3, 1 (16.0)	2, 1 (12.0)	9, 1 (40.0)
8	33	9, 4 (39.4)	2, 2 (12.1)	2, 1 (9.10)	13, 0 (39.4)
9	4	2, 1 (75.0)	—	—	0, 1 (25.0)
10	6	3, 1 (66.7)	—	—	1, 1 (33.3)
	102	40, 62.2^e^	9.2, 16.2	12.3, 8.1	38.5, 13.5

a C=cytoplasmic, N=nuclear.

b Number of cases separated by tissue microarrays (TA1,TA2).

c Non-neoplastic.

d Percentage of cases by Gleason score.

e Percentage of cases by individual tissue microarray.
